# The enduring enigma of sporadic chorea: A single center case series

**DOI:** 10.5334/tohm.800

**Published:** 2023-09-07

**Authors:** Pedro J. Garcia Ruiz, Lola Diaz Feliz, Cici E. Feliz, Isabel Lorda Sanchez, Almudena Avila Fernandez, Fiona Blanco Kelly, Maria Jose Trujillo Tiebas, Javier del Val, Inma Navas Vinagre

**Affiliations:** 1 Department of Neurology, Fundacion Jimenez Diaz, Madrid, Spain; 2 Department of Genetics, Fundacion Jimenez Diaz, Madrid, Spain

**Keywords:** Sporadic chorea, Hereditary chorea, Secondary chorea

## Abstract

Chorea can have a wide variety of causes including neurodegenerative, pharmacological, structural, metabolic, infectious, immunologic and paraneoplastic processes.

We reviewed the clinical records of patients with apparently sporadic choreic movements and no relevant family history, who presented to our neurology department (Hospital Fundación Jimenez Diaz) between 1991 and 2022.

We detected 38 cases of apparent sporadic chorea (ASC); Our analysis revealed 5 cases of genetic chorea (including 3 cases with Huntington’s disease) while 6 cases were autoimmune/hematological; 6 drug-related chorea, 5 metabolic-vascular, 5 due to miscellaneous conditions and 4 were of mixed etiology. No clear etiology was identified in 8 cases. The differential diagnosis of ASC is extensive and challenging.

**Highlights:**

- Chorea can have a wide variety of genetic and sporadic causes
- We reviewed the clinical records of patients with apparently sporadic chorea (ASC), who presented to our neurology department over the last 30 years
- We detected 38 cases of apparent ASC; Our analysis revealed a wide array of different sporadic conditions and 5 cases of genetic chorea
- The differential diagnosis of ASC is extensive and challenging

## Background

Chorea is characterized by the presence of fluctuating, brief, and unpredictable involuntary movements [1,2,3]. The diagnosis and management of chorea is frequently challenging, as the condition can be due to a wide variety of causes including neurodegenerative, pharmacological, structural, metabolic, infectious, immunologic and paraneoplastic processes [1,2,3,4,5,6,7,8,9]. In the absence of other apparent causes, ruling out Huntington’s disease (HD) is the first step in the diagnostic process [1,2,3,4,5,6,7,8,9,10]. Despite an extensive work-up, many cases remain undiagnosed, especially for those patients presenting with Apparent Sporadic Chorea ASC [4,7,8].

## Patients and Methods

We carried out a retrospective chart review of patients with ASC and no relevant family history, who presented to our neurology department from 1991 to 2022. We excluded patients with a positive family history. All patients underwent neuroimaging studies (brain CT or MRI scan). Laboratory tests included routine blood and urine analysis as well as blood tests for thyroid hormones, creatine phosphokinase, copper, ceruloplasmin and acanthocytes. Depending on the clinical context of each patient, autoimmune tests, coagulation studies, muscular biopsy and molecular analyses to exclude HD, chorea-acanthocythosis, hereditary ataxias, hereditary frontotemporal dementia or ADCY5-related dyskinesias were carried out. This study was approved by the Research Ethics Committee of our center.

## Results

Below we present the results of our research grouped by diagnosis.

### Genetic Chorea

Genetic chorea was diagnosed in 5 patients including 3 patients with late onset HD (cases 1–3), all presented mild late-onset chorea with typical oculomotor disturbances (CAG expansions 29-39-39 respectively). Also included in this group was a typical case of chorea-acanthocytosis (case 4) with progressive chorea and oral lesions, as well as a carrier of an ADCY5 mutation (case 5), who exhibited chorea-dystonia associated with ataxia. The patient had been initially referred to our department with a diagnosis of cerebral palsy (CP).

### Autoinmune and Haematological Cases

We detected several autoimmune/hematological cases (cases 6–10) including one female patient with juvenile Sydenham chorea (SC); a typical clear-cut case in a young female with asymmetric presentation (case 6). We studied 2 cases of lupus-related chorea, both with mild generalized chorea. The first patient (case 7) had a positive antiphospholipid antibodies; the second patient (case 8) presented nephrotic syndrome, arthritis, leukopenia and was positive for antinuclear antibodies. We also studied a patient with systemic vasculitis related with rheumatoid arthritis (case 9).

Finally, a patient with late onset chorea was diagnosed with JAK-mutation- positive- polycythemia vera (case 10). This patient presented with moderate generalized chorea and over time, she developed laboratory and clinical signs of polycythemia.

### Drug-related Chorea

This group (cases 11–16) comprised 6 patients, treated with antidopaminergic drugs (risperidone, olanzapine, clebopride, quetiapine, aripiprazole), opiates, antidepressants, or a combination of drugs (4 patients). One of these patients had chorea associated to other more typical aspects of tardive dyskinesia such as cranial dystonia. After drug withdrawal, four patients improved and no change was noted in the other two.

### Metabolic and Vascular

Two patients had chorea related to metabolic conditions (cases 17,18), both had uncontrolled diabetes and both improved with metabolic correction. Of note, chorea was the presenting symptom in case 18. Three other patients presented subacute asymmetric chorea following a vascular event (cases 19–21).

### Miscellanea and Mixed Ethiologies

We identified a case with classical mitochondrial disease presenting seizures, neurosensorial hearing loss, poor visual acuity, episodes of sudden hemiparesis and myopathy. Muscle biopsy disclosed typical ragged red fibers. We suspected the presence of mitochondrial encephalomyopathy, lactic acidosis, and stroke-like episodes (MELAS), although a molecular analysis of mtDNA ruled out MELAS, as well as myoclonic epilepsy with ragged red fibers, chronic progressive external ophthalmoplegia, and Kearns-Sayre syndrome; with the passage of time, this patient developed generalized chorea and cognitive decline (case 22).

We studied an intriguing case of a woman with renal failure who presented metformin-related severe chorea (case 23). The literature contains several reports of this etiology with similar clinical findings, probably due to severe metabolic acidosis (see discussion).

We also detected 2 cases of early onset chorea in patients with motor and cognitive static deficiency since early childhood (cases 24,25). CP-related chorea seems the best explanation.

Post-traumatic chorea was identified in one patient (case 26) who had a history of severe cranial trauma with extensive frontal lesions and presented with chorea and cognitive decline.

Four patients (case 27–30) had several etiologies in combination, such as vascular chorea associated with neuroleptic drug therapy; severe uncontrolled diabetes in addition to B12 deficit; vascular etiology combined with hepatic cirrhosis and chronic neuroleptic therapy in addition to long term depression and frontotemporal cognitive decline.

### Unknown Etiology

We were unable to establish a definite cause of chorea in 8 cases (cases 31–38), despite an extensive work-up. We suspected frontotemporal dementia in 4 cases (cases 30, 33,34 and 37) and hereditary ataxia in 2 patient (case 32 and 36) although the findings of genetic analyses were negative for hexanucleotide expansion (C9ORF72), progranulin mutation and/or common dominant hereditary/recessive ataxias (SCA 1,2-3-6,7,8, 17,36, DRPLA and Friedreich). We also suspected PMM2 mutation as a cause of adult-onset chorea plus ataxia and congenital cognitive decline (case 35), the most common congenital disorder of N-glycosylation, but genetic analyses did not permit such confirmation (only one confirmed PMM2 mutation). We also found the common haemochromatosis mutation in one case (case 38) in which the routine work-up disclosed elevated ferritin levels, the patient has been treated with periodic phlebotomy, although to date the chorea has not improved.

Table 1 summarizes the main results.

**Table 1:** Apparent Sporadic Chorea.


PATIENT	GENDER	AGE AT ONSET	PRESENT AGE	CLINICAL FEATURES	HD EXPANSION	PRIMARY DIAGNOSIS	ALTERNATIVE DIAGNOSIS	OTHER DATA

**1.**	M	80	88	1,2,4,8	29	HD		

**2.**	F	60	80	1,2,8	39	HD		

**3.**	M	75	79	1,2	39	HD		

**4.**	F	20	47	2,6,7	ND	CHOREA-ACANTHOCYTOSIS		

**5.**	F	5	39	2,3,6,7	ND	ADCY5		

**6.**	F	19	30	2,5	ND	SYDENHAM CHOREA		

**7.**	M	73	84	4	NEGATIVE	LUPUS		

**8.**	M	57	53	7	ND	LUPUS	ENOLISM	

**9.**	F	73	84	7	ND	SISTEMIC VASCULITIS, REUMATHOID ARTHRITIS		

**10.**	F	75	81	1,2	NEGATIVE	POLYCYTHEMIA		JAK 2 MUTATION

**11.**	M	77	85	0	NEGATIVE	DRUG-RELATED		

**12.**	F	79	82	3	NEGATIVE	DRUG-RELATED		

**13.**	F	76	82	2	NEGATIVE	DRUG-RELATED		

**14.**	F	64	70	2	ND	DRUG-RELATED		

**15.**	F	81	84	2	NEGATIVE	DRUG-RELATED		

**16.**	F	51	52	8	NEGATIVE	DRUG-RELATED		

**17.**	F	73	80	4	NEGATIVE	METABOLIC		

**18.**	F	90	91	5	ND	METABOLIC		

**19.**	M	83	84	5	ND	VASCULAR		

**20.**	F	83	84	5	ND	VASCULAR		

**21.**	M	62	68	5	NEGATIVE	VASCULAR		

**22.**	F	30	50	1,2,3,4,8	ND	MITOCHONDRIAL		DEAPHNESS

**23.**	F	58	60	4,8	ND	DRUG-RELATED	RENAL FAILURE	

**24.**	F	3	74	7	ND	CP		

**25.**	F	5	31	4,5,6	ND	CP	VASCULAR	

**26.**	M	52	59	1,2,4	NEGATIVE	POSTTRAUMATIC		MRI: FRONTAL LESION

**27.**	F	56	62	4,7,8	NEGATIVE	VASCULAR	DRUG-RELATED	

**28.**	F	80	83	4	NEGATIVE	VASCULAR	B12 DEFICIT	

**29.**	M	62	63	0	ND	METABOLIC	VASCULAR, LIVER SCIRROSIS	

**30.**	F	77	73	1,2,3,4,7,8	NEGATIVE	DRUG-RELATED	FRONTOTEMPORAL DEMENT	

**31.**	F	79	81	1,2	NEGATIVE	UNKNOWN	MULTIPLE MIELOMA	

**32.**	F	68	80	1,2,3	NEGATIVE	UNKNOWN	NEURODEGENERATIVE ATAXIA	

**33.**	F	74	76	1,2,3,4,8	NEGATIVE	UNKNOWN	FRONTOTEMPORAL DEMENT	

**34.**	F	73	75	1,2,4	NEGATIVE	UNKNOWN	FRONTOTEMPORAL DEMENT	

**35.**	F	40	53	1,2,3,4	NEGATIVE	UNKNOWN	PMM2 MUTATION	

**36.**	M	68	71	1,2,3	NEGATIVE	UNKNOWN	NEURODEGENERATIVE ATAXIA	

**37.**	M	56	58	1,2,4	NEGATIVE	UNKNOWN	FRONTOTEMPORAL DEMENT	

**38.**	M	55	58	1,2	NEGATIVE	UNKNOWN	HAEMOCHROMATOSIS	c2824 MUTATION


M: Male F: Female HD: Huntington’s Disease CP: Cerebral Palsy ND: Not Done; Clinical Features: 0: None 1: Oculomotor Disturbances 2: Motor Impersistence 3: Ataxia 4: Cognitive Decline 5: Asymmetric Chorea 6: Pyramidal Signs 7: Dystonia 8: Parkinsonism.

## Discussion

Five patients were diagnosed with hereditary chorea. This finding is hardly surprising since the absence of a family history does not rule out the presence of a genetic cause, including late-onset HD [2,3,6,8,11,12,13,14,15,16]. Our patients with HD (cases 1–3) had triplet expansion that fell within the intermediate range (29,39,39 triplets), range of repeat expansion in which many patients develop symptoms late in life [12,13,14,15,16]. Our patients did not recall any relatives having HD, as is frequently the case with late-onset HD [12,13,14,15,16].

We also detected a typical case of chorea-acanthocytosis (case 4) with progressive chorea and typical oral lesions. Oral lesions are critical red-flag for any patient presenting with chorea [4,5,6,7,8]. This case was successfully treated with deep brain stimulation as was reported elsewhere [17].

We evaluated a patient with an ADCY5 mutation presenting early-onset chorea associated with severe ataxia and dystonia. The patient was first studied many years before the mutation was described, and presented typical features of ADCY 5 mutation including chorea, axial hypotonia, and nocturnal bouts of severe dyskinesia [6,7,8,18].

Regarding the immune and hematological cases included in this series, we diagnosed a typical case of SC in a young woman who abruptly presented asymmetric chorea, in this particular case (previously the patient had been labelled as functional case) the presence of a hung-up knee jerk [19] prompted a diagnostic work-up. The disease course was favorable although some mild dyskinesias still appeared during stress. In addition, she recently had a recurrence of chorea during her first pregnancy. Nowadays, SC remains an important cause of chorea in younger adults [20,21].

Three patients were diagnosed with lupus-related and vasculitis-related chorea. Chorea may be a classic, albeit rare, manifestation of lupus [8,10,22], but it can also occur as part of other immunologic syndromes including Sjogren’s syndrome and rheumatoid arthritis [8,10,23,24,25,26].

Polycythemic chorea should be considered in the differential diagnosis of late-onset chorea [8,10,27]. Chorea was the initial manifestation in our patient, a JAK mutation carrier (case 10). This rare, but potentially treatable chorea must be kept in mind, especially for late-onset chorea in females.

Chorea may be a side effect of many drugs especially neuroleptics, but also, less commonly, anticonvulsants, anticholinergics, antidepressants and opioids [8,10,28,29,30,31]. Four out of 6 patient were taking several drugs. Of note, patients are frequently unaware that this type of drugs must be monitored and eventually withdrawn; as is the case with antiemetic and or anti-dizziness medication [28]. In any case, careful review of the full medication must be performed in any patient presenting with movement disorders, including chorea. Four patients improved after drug discontinuation but only one reached a complete remission.

Chorea may be observed in many systemic and metabolic conditions such as uncontrolled diabetes, liver and renal disease, hyperthyroidism, electrolyte disturbances and vitamin B12 deficiency [8,10,32,33,34,35,36,37,38]. Suspicion of metabolic disorders can be especially high when movement disorders, including chorea, are observed in emergency settings [8,10]. Chorea associated with nonketotic hyperglycemia is a well-recognized syndrome characterized by the acute occurrence of hemichorea in elder diabetes II patients [8,10,36,37]. Frequently, but not always, brain MRI shows signs (T1 hyperintensity) of the so-called ¨diabetic striatopathy¨ [36,37,38]. Of note, Hemichorea/hemiballism associated with nonketotic hyperglycemia can be the presenting manifestation of diabetes mellitus [36,37].

Vascular conditions such as ischemic and hemorrhagic strokes can be associated with most movement disorders including chorea. Acute occurrence of subthalamic-related hemichorea or hemiballismus is rarely overlooked in emergency settings, but many other brain areas may be also involved including the putamen, pallidum, thalamus, caudate nucleus, corona radiata and even cortical areas [39,40,41,42,43,44]. Suri et al [43] suggested that chorea was the earliest post-stroke movement disorder, whereas dystonia and tremor manifested several months after the stroke.

The subset covering miscellaneous cases and those with mixed etiology comprises 9 patients in this series. We had the opportunity to follow a patient with typical mitochondrial symptoms such as seizures, neurosensorial hearing loss, poor visual acuity, episodes of sudden hemiparesis, and myopathy; muscle biopsy confirmed the presence of ragged red fibers, but genetic analyses ruled out MELAS, MERF and KSS. Over time, the patient developed progressive chorea. Mitochondrial diseases may manifest a plethora of movement disorders including chorea [8,9,45].

Dyskinetic cerebral palsy is the second most common type of CP after spastic forms [46]. Chorea is very frequently associated with dystonia and is rarely the primary source of disability [46,47]. In any case, caution must be taken before attributing any chronic movement disorder of pediatric onset to CP, since many genetic mimics exist [48], including the recently described ADCY 5 mutation [6,7,8,18].

We also studied a very rare case of a patient with chronic renal insufficiency who developed severe chorea associated with metformin intake. There are several reports of metformin-induced chorea [34], likely due to severe metabolic acidosis [34,49].

A man who had a severe traumatic head injury following a 10-meter fall with frontal lesion presented frontal lobe disorder and chorea. Post-traumatic movement disorders are well known, and chorea is a rare complication of head injury [50,51,52,53].

We were unable to establish a definitive diagnosis for 8 patients, although we suspected degenerative ataxias in 2 and frontotemporal dementia in 4 patients. Frontotemporal dementia may be associated with chorea [54,55,56,57,58], but it is worth recalling that an ample majority of patients with non-familiar frontotemporal dementia do not harbor genetic pathogenic variants according to recent case series [58].

Finally, movements disorders associated with haemochromatosis, including chorea, have been described [59], but this apparent association has been debated [60] since haemochromatosis is a frequent condition. For now, this case is still under study.

To date, two interesting case series of sporadic chorea have been published [3,9], both from Italy. Despite certain differences with our series, it seems that vascular, metabolic and drug-related causes explain most cases of ASC. Interestingly, according to Piccolo et al [3], infectious etiology represented a substantial fraction of cases. Here it must be noted that AIDS and/or AIDS-related infections were associated with hyperkinesias several decades ago, when antiviral AIDS agents were not yet widely used [3].

## Conclusion

In summary, the differential diagnosis of ASC is extensive and challenging, even though the etiology of acute or subacute chorea may be suspected rapidly. In such cases, structural, infectious, metabolic, or drug-induced chorea are the main candidates. However a proportion of genetic choreas may appear with no known family history, including HD (with small triplet expansions), recessive choreas such as chorea-acanthocytosis, and/or recently described choreas such as those due to an ADCY5 mutation [1,2,4,5,6,7]. In any case, a significant proportion of both ASC and genetic choreas have no apparent etiology at present [1,2,3,4,5,6,7,8,9]. and probably other, currently unknown mutations are responsible, especially those related to frontotemporal dementia.
